# New insights into the pathogenesis of SARS-CoV-2 during and after the COVID-19 pandemic

**DOI:** 10.3389/fimmu.2024.1363572

**Published:** 2024-06-07

**Authors:** Jonatan J. Carvajal, Valeria García-Castillo, Shelsy V. Cuellar, Claudia P. Campillay-Véliz, Camila Salazar-Ardiles, Andrea M. Avellaneda, Christian A. Muñoz, Angello Retamal-Díaz, Susan M. Bueno, Pablo A. González, Alexis M. Kalergis, Margarita K. Lay

**Affiliations:** ^1^ Department of Biotechnology, Faculty of Marine Sciences and Biological Resources, University of Antofagasta, Antofagasta, Chile; ^2^ Department of Pharmacology, Faculty of Biological Sciences, University of Concepción, Concepción, Chile; ^3^ Center for Research in Physiology and Altitude Medicine (FIMEDALT), Biomedical Department, Faculty of Health Sciences, University of Antofagasta, Antofagasta, Chile; ^4^ Department of Basic Sciences, Faculty of Sciences, Universidad Santo Tomás, Antofagasta, Chile; ^5^ Research Center in Immunology and Biomedical Biotechnology of Antofagasta (CIIBBA), University of Antofagasta, Antofagasta, Chile; ^6^ Department of Medical Technology, Faculty of Health Sciences, University of Antofagasta, Antofagasta, Chile; ^7^ Millennium Institute on Immunology and Immunotherapy, Department of Biotechnology, Faculty of Marine Sciences and Biological Resources, Department of Medical Technology, Faculty of Health Sciences, University of Antofagasta, Antofagasta, Chile; ^8^ Millennium Institute on Immunology and Immunotherapy, Facultad de Ciencias Biológicas, Pontificia Universidad Católica de Chile, Santiago, Chile; ^9^ Departamento de Endocrinología, Facultad de Medicina, Pontificia Universidad Católica de Chile, Santiago, Chile

**Keywords:** SARS-CoV-2, COVID-19, pathogenesis, immune response, pathophysiology, variants, host factors, long-COVID-19

## Abstract

Severe acute respiratory syndrome coronavirus 2 (SARS-CoV-2) is responsible for the respiratory distress condition known as COVID-19. This disease broadly affects several physiological systems, including the gastrointestinal, renal, and central nervous (CNS) systems, significantly influencing the patient’s overall quality of life. Additionally, numerous risk factors have been suggested, including gender, body weight, age, metabolic status, renal health, preexisting cardiomyopathies, and inflammatory conditions. Despite advances in understanding the genome and pathophysiological ramifications of COVID-19, its precise origins remain elusive. SARS-CoV-2 interacts with a receptor-binding domain within angiotensin-converting enzyme 2 (ACE2). This receptor is expressed in various organs of different species, including humans, with different abundance. Although COVID-19 has multiorgan manifestations, the main pathologies occur in the lung, including pulmonary fibrosis, respiratory failure, pulmonary embolism, and secondary bacterial pneumonia. In the post-COVID-19 period, different sequelae may occur, which may have various causes, including the direct action of the virus, alteration of the immune response, and metabolic alterations during infection, among others. Recognizing the serious adverse health effects associated with COVID-19, it becomes imperative to comprehensively elucidate and discuss the existing evidence surrounding this viral infection, including those related to the pathophysiological effects of the disease and the subsequent consequences. This review aims to contribute to a comprehensive understanding of the impact of COVID-19 and its long-term effects on human health.

## 1 Introduction

In December 2019, a novel coronavirus was identified by sequencing nasopharyngeal samples from patients experiencing severe pneumonia in Wuhan City, China. Initially designated as “2019-nCoV,” subsequent sequence analysis classified it as severe acute respiratory syndrome coronavirus 2 (SARS-CoV-2). This virus is the cause of Coronavirus Acute Respiratory Disease 2019 (COVID-19). Due to its heightened transmissibility and asymptomatic cases, SARS-CoV-2 rapidly disseminated globally, prompting its declaration as a pandemic on March 11, 2020 (1–3). This virus enters the body through the upper respiratory tract, mainly affecting the lungs. However, this pathogen can affect different systems, such as the gastrointestinal, renal, and Central Nervous Systems (CNS). Four years after the first reported case, the World Health Organization (WHO) has notified more than 771,820,937 confirmed cases; 6,978,175 deaths due to SARS-CoV-2 infection; and 13,534,474,309 doses of COVID-19 vaccines distributed globally. The fatality rate of SARS-CoV-2 is estimated to be between 1 to 5%, which is significantly lower when compared to the rate of severe acute respiratory syndrome coronavirus 1 (SARS-CoV-1) and the Middle East Respiratory Syndrome coronavirus (MERS-CoV), which are 9.7% and 34%, respectively (4).

Most recently, in May 2023, the World Health Organization (WHO) declared “The end of COVID-19 as a public health emergency.” This declaration signifies a substantial global improvement in the situation. However, it’s crucial to note that SARS-CoV-2 persists actively within the population, leading to ongoing transmission and the unfortunate loss of human lives (5, 6). Nonetheless, this WHO statement does not eliminate the possibility of experiencing future COVID-19 pandemic events, which underlines the need for continued surveillance and preparation for possible epidemic outbreaks or pandemics caused by SARS-CoV-2 in the future.

Coronaviruses (CoV) are classified within the order *Nidovirales*, suborder *Cornidovirineae*, family *Coronaviridae*, and subfamily *Orthocoronavirinae*. This subfamily is further divided into four genera: *Alphacoronavirus* (αCoV), *Betacoronavirus* (βCoV), *Gammacoronavirus* (γCoV), and *Deltacoronavirus* (δCoV). SARS-CoV-2, the causative agent of COVID-19, falls into the βCoV genus, sharing this classification with other clinically significant viruses, including SARS-CoV and MERS-CoV (6–9).

Over the last century, the emergence of several clinically relevant CoVs has had a dramatic impact on public health. Furthermore, their ability to “cross host barriers” and infect various organisms poses a future risk that requires careful consideration. Cross-species transmission can occur due to proximity between organisms, shared ecological niches, predation, or interspecies contact. Another mechanism facilitating the transmission across host barriers is the occurrence of random mutations in binding receptors. Transmission from more than one host also occurs in CoVs, such as MERS-CoV (10). Understanding the dynamics and mechanisms behind these cross-species transmissions is crucial to proactively assessing and mitigating the risk of new diseases or pandemics derived from CoVs (9, 11, 12).

The origin of SARS-CoV-2 remains unknown. There are multiple hypotheses about it, some of which are as follows: 1) SARS-CoV-2 comes from a mutation of the bat coronavirus RaTG13, which shares 96.2% similarity of its genome. It is thought that this virus mutated due to constant interaction with humans (predation of bats by humans), which forced it to adapt and thus be able to infect this new host; 2) SARS-CoV-2 comes from the mutation of human SARS-CoV (which would explain its ability to infect humans naturally) and through a series of random mutations resulting from selective and immunological pressures. In this scenario, it is thought that SARS-CoV mutated as a defense mechanism against these pressures, giving rise to a new type of virus. Finally, SARS-CoV-2 comes from the Malayan pangolin, with a similarity of 85.5 to 92.4%. Similar to the case of the bat hypothesis, the virus adapted to this new host due to the constant predation by humans against these animals. In summary, the exact origin of SARS-CoV-2 remains to be elucidated, making it difficult to determine whether the origin of this viral infection is zoonotic or abiotic. The ongoing investigation into these theories is essential for a comprehensive understanding of the virus’s origins and potential implications for public health (10–16).

This new infectious agent uses its Receptor Binding Domain (RBD) to bind, with high affinity, to Angiotensin Converting Enzyme 2 (ACE2) and Type II Transmembrane Serine Protease Coreceptor (TMPRSS2) to enter the host cells. The importance of ACE2 arises from the fact that it is an enzyme expressed in various organs and at different quantities (11). This enzyme plays a fundamental role in regulating blood pressure, kidney function, and electrolyte balance. In short, it is essential to maintain physiological homeostasis (17). In the context of infection, SARS-CoV-2 is associated with high levels of ACE2 (the higher the concentration of this enzyme, the greater the susceptibility to infection) (18). ACE2 can be elevated due to stress factors, low blood pressure, diet, or metabolic disorders (18, 19). Understanding the physiological effects of ACE2 concentrations in the body is crucial to elucidate the pathophysiological consequences of SARS-CoV-2 infection and its systemic effects.

The symptoms of COVID-19 are many and vary significantly among individuals. However, initial manifestations typically include fever, anosmia, and ageusia (20, 21). As SARS-CoV-2 infection progresses, additional signs and symptoms such as myalgia, fatigue, nasal congestion, sore throat, rash, and diarrhea may manifest. However, the latter set of signs and symptoms are comparatively less common (22). Despite COVID-19 being a disease with a mortality rate lower than SARS-CoV-1, the severity of the illness differed significantly from person to person. For some individuals, it presented as a mild cold, while for others, it posed a severe threat to their lives. Initial reports indicated that children and young adults with COVID-19 were often asymptomatic, while older adults experienced more severe forms of the disease. Notably, the presence of comorbidities increased the risk of death among individuals affected by COVID-19 (5, 23). Understanding the diverse clinical presentations of COVID-19 is essential for effective management and public health interventions.

As SARS-CoV-2 continues to circulate, the emergence of variants poses an ongoing health challenge. Each variant exhibits unique characteristics, contributing to distinct clinical courses, increased immune resistance, or heightened efficiency in dissemination (**Table 1**). These variations among strains underscore the dynamic nature of the virus and the importance of continuous monitoring and research to understand and address the evolving landscape of the pandemic (68, 69).

**Table 1:** List of SARS-CoV-2 variants of concern worldwide.

Currently circulating VOCs							
Variant.	Estimated Date of emergency/ Location.	Spike mutations of interest (https://covdb.stanford.edu/page/mutation-viewer/#sec_alpha; accessed on 9 April 2024.	Main Concerns				
			Transmissibility.	Disease severity.	Vaccine efficacy.	Neutralizing Abs efficacy MAbs Therapy.	Spike mutations involved in immune evasion/virulence.
Omicron BA.1 o B.1.1.529	24 of November 2021/ South Africa.	A67v, Δ69/70, T95I, G142D, Δ143/145, N211I, Δ212/212, R214ins, G339D, S371L, S373P, S375F, K417N, N440K, G446S, S477N, T478K, E484A, Q493R, G496S, Q498R, N501Y, Y505H, T547K, D614G, H655Y, N679K, P681H, N764K, D796Y, N856K, Q954H, N969K, L981F.	More transmissible than Beta and Delta variants (24).	It has been reported that the risk of hospitalization or death was 65% lower compared to cases with the Delta variant, and the risk of ICU admission was 83% lower (25).	BNT162b vaccine show a high level of protection with three doses. Between 20% and 24% of those vaccinated with BNT162b2 had detectable neutralizing antibodies against Omicron variants However (26), they were not detected in those vaccinated with Coronavac (26).	The monoclonal antibody Imdevimab lost activity against Omicron, while Casirivimab, Tixagevimab and Cilgavimab maintained neutralizing activity against this variant.	Pseudovirus experiments and structural modeling indicated that mutations T478K (27), 501Y (28), and D614G (28), could increase the affinity and tightness of binding with ACE-2 receptor, increasing the infectivity of this variant.
Omicron subvariant BA.1.1	November 2021/Botswana and South Africa.	A67V, Δ69/70, T95I, G142D, Δ143-145, N211I, Δ212, R214ins, G339D, R346K, S371L, S373P, S375F, G446S, S477N, T478K, E484A, Q493R, G496S, Q498R, N501Y, Y505H, T547K, D614G, H655Y, N679K, P681H, N764K, D796Y, N856K, Q954H, N969K, L981F.	The Omicron sub‐variants (BA.1.1, BA.2 and BA.3) are likely more transmissible than omicron (BA.1) and Delta (29).	People infected with Omicron variants BA.1 and BA.2 are similarly less likely to be hospitalized compared with those infected with Delta variant (30).	Two doses of BNT162b2 did not elicit robust neutralization against this subvariant (31).	Sotrovimab retained activity against BA.1 subvariant (32, 33).	The Omicron sub-variants shared 11 common mutations G339D, S373P, S375F, K417N, N440K, S477N, T478K, E484A, Q493R, Q498R, and N501Y in RBD that may contribute significantly to changing the immune evasion (29).
Omicron subvariant BA.3	November 2021/South Africa.	A67v, Δ69/70, T95I, G142D, Δ143-145, N211I, Δ212/212, D614G, H655Y, N679K, P681H, N764K, D796Y, Q954H, N969K.	BA.2 and BA.3 subvariants are more transmissible than Omicron BA.1 and Delta (34, 35).	There is no evidence.	BA.3 spike protein evades the BNT162b2-elicited neutralization more efficiently than BA.1 and BA.2 subvariants (31).	There is no evidence.	H655Y, N679K and P681H mutations could increase spike cleavage and facilitate virus transmission (34).
Omicron subvariant BA.2	November 2021/South Africa.	T19I, L24S, Δ25-27, G142D, V213G, G339D, S371F, S373P, S375F, T376A, D405N, R408S, K417N, N440K, S477N, T478K, E484A, Q493R, Q498R, N501Y, Y505H, D614G, H655Y, N679K, P681H, N764K, D796Y, Q954H, N969K.	Unvaccinated individuals had a higher transmissibility with BA.2 compared to BA.1 (36). BA.2 and BA.3 subvariants are more transmissible than Omicron BA.1 and Delta (35).	Risk ratios of hospitalization with BA.1 vs BA.2 indicate non-significant differences between individuals infected with BA.1 and BA.2 for sex, age, reinfection or 30-day mortality (37).	The evidence of the short-term effectiveness against Omicron infection of a third or fourth dose of either the mRNA or inactivated vaccine, in terms of cumulative infection attack rate (IAR), BA.2 vaccine effectiveness was 45% (38).	The monoclonal antibody Cilgavimab efficiently neutralizes BA.2 subvariant (32).	The Omicron sub-variants shared 11 common mutations G339D, S373P, S375F, K417N, N440K, S477N, T478K, E484A, Q493R, Q498R, and N501Y in RBD that may contribute significantly to changing the immune evasion (29).
Omicron subvariant BA.4	January 2022/South Africa.	T19I, L24S, Δ25/27, Δ69/70, G142D, V213G, G339D, S371F, S373P, S375F, T376A, D405N, R408S, K417N, N440K, L452R, S477N, T478K, E484A, F486V, Q498R, N501Y, Y505H, D614G, H655Y, N679K, P681H, N764K, D796Y, Q954H, N969K.	BA.4 and BA.5 may be more transmissible than the other Omicron variants (39). Spike region mutations at positions L452R, F486V, R493Q, are responsible for the increased spread and transmissibility of the virus (40).	There is no evidence.	Mutations on BA.4/5 are significantly (4.2‐fold) more resistant, and as a result, it has a greater potential to produce vaccine breakthrough infections (an infection of a fully vaccinated person) (41).	The therapeutic antibodies Bebtelovimab and Cilgavimab can effectively neutralize BA.4 and BA.5. (42)	L452R and F486V mutations could cause increased escape to antibodies (43).
Omicron subvariant BA.5	February 2022/South Africa	T19I, L24S, Δ25/27, Δ69/70, G142D, V213G, G339D, S371F, S373P, S375F, T376A, D405N, R408S, K417N, N440K, L452R, S477N, T478K, E484A, F486V, Q498R, N501Y, Y505H, D614G, H655Y, N679K, P681H, N764K, D796Y, Q954H, N969K.	BA.4 and BA.5 may be more transmissible than the other Omicron variants (39).	There is no evidence..	Mutations on BA.4/5 are significantly (4.2‐fold) more resistant, and as a result, it has a greater potential to produce vaccine breakthrough infections (an infection of a fully vaccinated person) (41).	The therapeutic neutralizing antibodies Bebtelovimab and Cilgavimab can effectively neutralize BA.4 and BA.5 (42).	L452R and F486V mutations could cause increased escape to antibodies (43).
**Previously circulating VOCs**							
Alpha B.1.1.7	February 2020/ United Kingdom	Δ69/70, Δ144/144, N501Y, A570D, D614G, P681H, T716I, S982A, D1118H.	5% more transmissible than previous variants (44). Estimated increase in transmissibility of 29% compared to non-VOC variants (45). Alpha VOC has a 43%-90% higher reproduction number compared to preexisting variants (46).	It was associated with a 73% higher risk of death and a 62% higher risk of hospital admission compared to wild-type virus (35).	After a first and second vaccination with the BNT162b2 vaccine (Pfizer-BioNTech), a modest reduction in neutralizing activity against this variant was observed (34). Novavax clinical trials showed 85.6% effectiveness against this variant (47).	Neutralizing monoclonal antibodies to the RBD or N-terminal domain demonstrated diminished activity against this variant (47, 48).	N501Y mutation increases the binding affinity to ACE-2 receptor and, thus, the transmissibility (49, 50). Also, is associated with the escape of neutralizing antibodies (51, 52). In a mouse-adapted strain model, N501Y mutation favors the interaction with ACE2 receptor and promotes the virus entry (53).
Beta B.1.351	December 2020/ South Africa.	D80A, D215G, Δ241/243, K417N, E484K, N501Y, D614G, A701V.	Estimated 25% increase in transmissibility compared to non-VOC variants (45). Estimated increase in transmissibility of 50% compared to previous circulating variants.	Beta variant cases have significantly higher hospitalization rates compared to other variants (54).	The effectiveness of the mRNA-1273 (Moderna) vaccine against this variant was 96.4% after the second dose (55). Novavax clinical trials showed 60% effectiveness against this variant (47).	Beta variant can completely evade neutralization by mAb LY-CoV55 (52).	E484K mutation is associated with increased binding to ACE-2 receptor in this variant (56).
Gamma P.1	6 January 2021, Brazil	L18F, T20N, P26S, D138Y, R190S, K417T, E484K, N501Y, D614G, H655Y, T1027I, V1176F.	Estimated increase in transmissibility of 38% compared to non-VOC variants (45). 1.7 to 2.4 times more transmissible than previous circulating variants (57).	Gamma variant cases had significantly higher ratios of hospitalization and ICU admission compared to non-VOC cases (54).	This variant was reported to be more resistant to neutralization by serum collected from individuals who received NBT162b2 or mRNA-1273 vaccines (58). Reduced serum ability to neutralize post-vaccination with ChAdOx1 vaccine (59).	A study reported that this variant is not only refractory to multiple monoclonal antibodies, but also more resistant to neutralization with convalescent plasma (58).	Mutations K417T, E484K and N501Y has been associated with an increased ACE-2 receptor binding affinity (56), transmissibility (60), and resistance to immune response (56).
Delta B.1.617.2	October 2020/ India	T19R, E156G, Δ157/158, L452R, T478K, D614G, P681R, D950N.	Estimated increase in transmissibility of 97% compared to non-VOC variants (45).	It was associated with a higher oxygen requirement, ICU admission, and death compared to other VOCs, as well as significantly higher viral loads (61).	A reduced ability of serum to neutralize this variant was reported post vaccination with BNT162b2 vaccine (62–64). Administration of two doses of the BNT162b2 or ChAdOx1 nCoV-19 vaccines generated a neutralizing response in 95% of individuals, with titers three to five times lower than the alpha variant (65).	A study reported that this variant is resistant to neutralization by some anti-N-terminal and anti-RBD monoclonal antibodies, such as Bamlanivimab (51).	L452R mutation increase the interaction between RBD, the ACE-2 receptor and infectivity (66). T478K and L452R mutation helps to stabilize the RBD-ACE-2 complex and increases the infectivity rate of the virus (67). P681R mutation has been associated with an increased transmissibility and viral load (67).

Although the clinical course of the infection by SARS-CoV-2 varies from person to person, it has been reported that after COVID-19, sequelae of varying durations range from one week to prolonged periods, possibly lifetime (70, 71). Although the mechanism by which these sequelae originate is not entirely clarified, it has been postulated that they may occur due to: 1) direct damage of the virus to the tissue or cells; 2) cellular damage caused by the immune system when fighting the infection; 3) direct damage to tissues resulting from a deregulation of the immune response; or 4) metabolic alterations produced by the SARS-CoV-2 infection. This phenomenon has been described in the literature with various terms, “Long-COVID-19”, “long-term COVID-19”, “post-acute COVID-19 syndrome (PACS)” or “chronic COVID-19”. For consistency, this review adopts the terminology proposed by the World Health Organization (WHO), referring to these conditions as “Long-COVID-19” (72, 73).

This review aims to delineate the viral and host components that facilitated SARS-CoV-2 infection and influenced the severity of COVID-19 during the pandemic. Additionally, the focus will be on elucidating the significance of “Long-COVID-19,” exploring distinctions between variants, examining various immunization strategies to combat this pathogen, and evaluating host conditions contributing to infection severity in the post-pandemic period (74–77). Given the challenges posed by the high transmission rate, immune evasion, and mutation rate of SARS-CoV-2, it remains uncertain whether the virus will evolve into an endemic state. Consequently, sustained vigilance and continued research efforts are imperative to mitigate the severity of infections, reduce mortality rates, and address potential long-term effects (78).

## 2 SARS-CoV-2 infection onset, progression, and pathophysiology of COVID-19

The initiation of infection and the progression of the pathophysiology of COVID-19 have been the focus of numerous investigations. Some studied characteristics of SARS-CoV-2 focus on its resistance to environmental pressures, the affinity of its receptor to ACE2, and the general clinical course. Nevertheless, significant gaps in our understanding of this agent persist, emphasizing the need for further research to uncover additional information.

### 2.1 SARS-CoV-2 and its infection cycle

**Figure 1:**
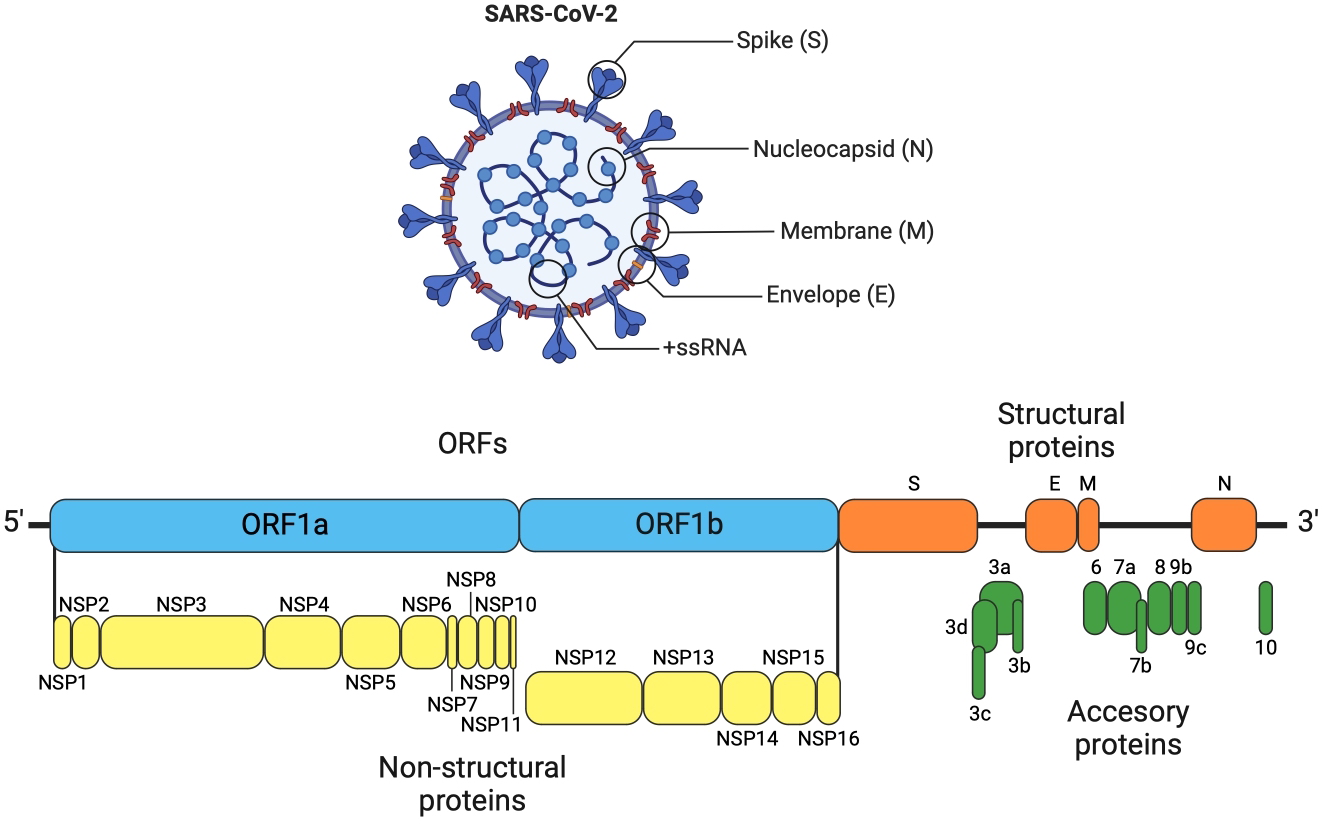
SARS-CoV-2 genome organization. The genome of SARS-CoV-2 is organized similarly to other coronaviruses, consisting of the following elements: a 5´ untranslated region, ORF1a, which encodes two overlapping polyproteins (pp1a and pp1ab). The pp1a non-structural protein corresponds to nsp1 to nsp10, while pp1ab non-structural protein comprises nsp12 to nsp16, which are further cleaved into 16 non-structural proteins (nsp1–16). The 3´ untranslated region contains the structural genes that encode four structural proteins: Spike Surface glycoprotein (S), Envelope glycoprotein (E), Nucleocapsid (N), and main protein or Matrix (M). The accessory genes are interspersed among the structural genes (79, 80).

**Figure 2:**
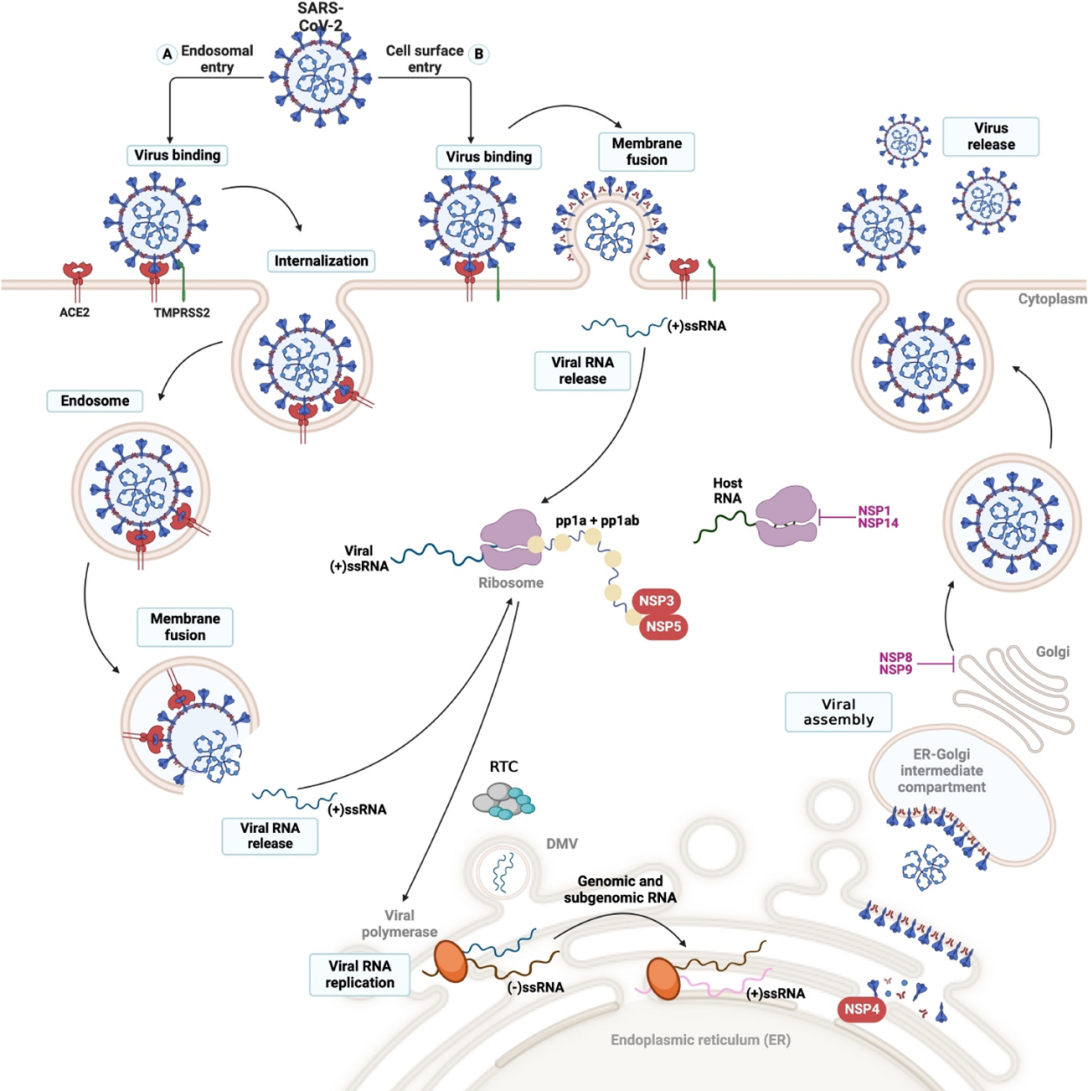
SARS-CoV-2 cell entry and viral cycle. The viral cycle commences with the binding of the Spike (S) protein to the ACE-2 receptor, facilitated by either cell surface or endosomal entry. (2) The TMPRRS2 protease (transmembrane protease serine) mediates the fusion of the virus-cell membranes through the cleavage of protein S, allowing the virus to enter the cytosol of the host cell. (3) Once internalized, the virus undergoes uncoating, releasing the viral genome into the cytosol, where it undergoes replication and translation. (4) This process generates two polyproteins: pp1a and pp1b, which are subsequently cleaved by the viral protease present in the nonstructural proteins (nsps) encoded by the virus. (5) In endoplasmic reticulum (ER)-derived double-membrane vesicles (DMVs), the negative-stranded genome serves as a template to generate the entire positive-stranded genome and subgenomic RNA (sgRNA). Translation of sgRNA in the ER results in the synthesis of the structural glycoproteins N, S, M, and E, which are utilized for viral assembly in the ER-Golgi intermediate compartment (ERGIC). (6) Finally, the entire positive-stranded genome is encapsulated in newly synthesized virions, which are released from the cell via exocytosis.

**Figure 3:**
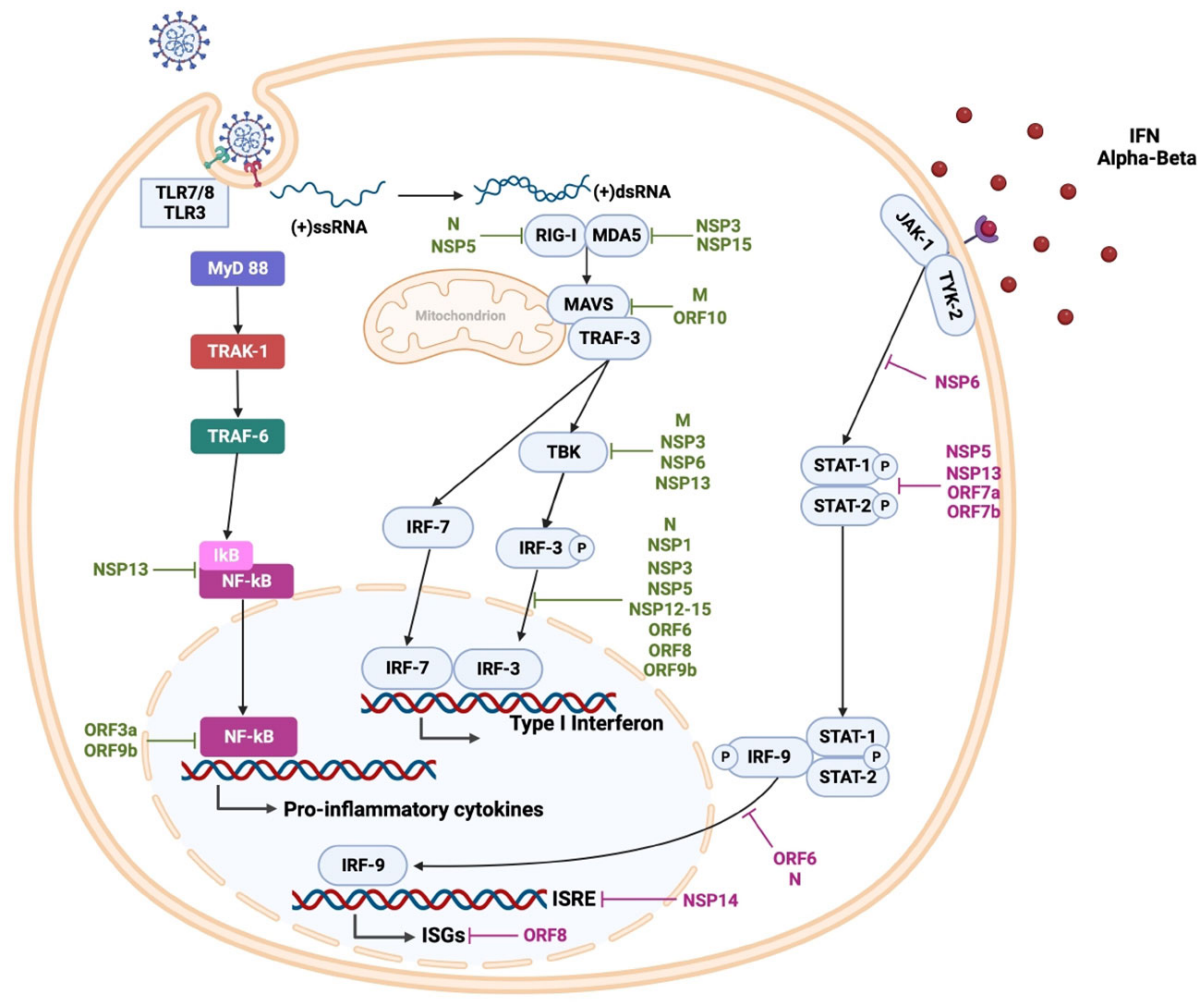
SARS-CoV-2 innate immune response and viral evasion. During viral replication, cytosolic double-stranded RNA is detected by retinoic acid-inducible gene I (RIG-1) and melanoma differentiation-associated protein 5 (MDA5). Viral genomic single-stranded RNA is detected by toll-like receptor (TLR) 7 and TLR8 in the endosomes of plasmacytoid dendritic cells and B cells, as well as myeloid cells, respectively. Additionally, endosomal TLR3 recognizes double-stranded RNA in various cells, and TLR4 participates in the detection of oxidized phospholipids (OxPLs) induced by SARS-CoV-2 infection. These interactions lead to the activation of downstream transcription factors, most notably IRF3 and NF-κB, promoting the expression of type I and III interferons (IFNs) and proinflammatory cytokines such as TNF-α, transforming Growth Factor β (TGF-β), interleukins (IL-1β, IL-6, IL-8, IL-12, IL-18), and chemokines like chemokine (C-C motif) ligand 2 (CCL2), CCL3, CCL5, C-X-C motif chemokine ligand 8 (CXCL8), CXCL9, CXCL10. Furthermore, endosomal TLR3 can recognize double-stranded RNA in various cells, while TLR4 detects oxidized phospholipids (OxPL) induced by SARS-CoV-2 infection. SARS-CoV-2 antagonizes the innate immune response at different steps; specific viral proteins interfere with type I IFN production (shown in green) and interferon signaling (shown in purple), impairing the early antiviral state.

SARS-CoV-2 has a single-stranded positive-sense RNA genome (ssRNA+) with an approximate size of 29,891 nucleotides (~30 kb), containing 15 ORFs (**Figure 1**). During the infection process, replication, and virulence of SARS-CoV-2, different viral proteins intervene, such as structural proteins (SP) and non-structural proteins (NSP). Both kinds of proteins play multiple roles and functions, which are shown in the **Table 2** and **Figures 1**–**3** (163, 164).

**Table 2:** Functions of SARS-CoV-2 proteins related to viral pathogenicity.

Structural proteins	
M	Inhibits the innate antiviral response by disrupting the recruitment of TRAF3, TBK1, and interferon regulatory factor (IRF3) to the MAVS complex (81).
	Viral suppressor of viral RNA interference, through RNA binding (82). Prevents activation of the RNA sensor RIG-I and inhibits nuclear translocation of IRF3 (83). Affects the recognition of dsRNA by RIG-I. Reduces the formation of antiviral stress granule, interacting with G3BP1, which is critical to cGAS-STING and RIG-I/MDA5 signaling pathways (84). Affects the signal transducer and activator of transcription (STAT1/STAT2) nuclear translocation impairing interferon signaling (85). Package genomic RNA during virion assembly (86).
S	Binds to ACE-2 receptor through the RBD. It has a relevant role in attachment, fusion, and entry to the host cells (87).
E	Relevant for viral assembly and budding (88). It contributes to epithelial damage and pathogenesis through its viroporin channel properties. It is suggested to bind zonula occludens -1 (ZO-1) leading to epithelial barrier compromise (89).
Nonstructural proteins	
NSP1	Binds to the 40S ribosomal subunit, competing with (messenger RNA (mRNA). Viral mRNA is favored, then it inhibits the host´s cellular translation machinery. Then it blocks RIG-I dependent innate immune response (90). Reduces IRF3 translocation into the nucleus blocking type I IFN induction (91). Inhibits NLRP3 inflammasome activation in an early stage of SARS-CoV-2 infection (92). Inhibits IFN-β-induced ISRE promoter activity (93).
NSP2	It has a stabilizing mutation in the endosome-associated-protein-like domain that may be responsible of SARS-CoV-2 high ability of contagious (94). N terminal domain is a novel zinc finger domain consisting of three zinc fingers. May be involved in binding nucleic acids and regulating intracellular signaling pathways (95). It is suggested to link viral transcription in the RTC to the translation initiation (96).
NSP3	Processes the polyprotein (papain protease activity), then it is essential in viral replication (97). Contains a functional SARS-Unique Domain (SUD) domain that binds human Paip1, favoring viral protein synthesis (98). SUD domain also binds guanine quadruplexes (99). This interaction may modulate guanine quadruplexes dynamics in viruses and host genomes, being relevant in SARS-CoV-2 pathogenesis (100). A Central component of the molecular pore of the double membrane of coronavirus replication organelle (101). Antagonizes ISG15- dependent activation of MDA-5 (102). PLpro Contributes to the cleavage of ISG15 from IRF3, decreases the phosphorylation of TBK-1 and nuclear translocation of IRF3 (103). A macrodomain of NSP3 hydrolyzes ADP-ribose modifications (product of IFN-type I and II signaling) (104).
NSP4	Produces changes in ER structure (105), helping to the ensambling of the RTC and viral replication (106). Membrane protein potential component of the pore in double-membrane vesicles (DMV), a pathway used by coronavirus RNA products to reach the cytosol (101).
NSP5 (3CLpro or Mpro)	Main protease (MPro). Plays a relevant role in SARS-CoV-2 replication, cleaving viral polyprotein. This ability is stronger in SARS-CoV-2 if it is compared with SARS-CoV NSP5, suggesting faster replication (107). Impairs host trafficking pathways. Interacts with histone deacetylase 2, inhibiting its mobilization to the nucleus, and with tRNA methyltransferase 1, removing its zinc finger and nuclear localization signal (108). Inhibits IFN type I induction through cleaving RIG-I, which loses its ability to activate MAVS. It also promotes ubiquitination and proteasomal degradation of MAVS (107). Impairs nuclear translocation of phosphorylated IRF3, suppressing type I IFN response (109). Antagonizes cGAS-STING activity (110). Reduces the formation of antiviral stress granules (84). Inhibits JAK-STAT signaling impairing ISG induction. It is suggested that Mpro may promote the degradation of STAT1 (111). 3CLpro cleaves NLRP12 and TAB1. NLRP12 cleavage might explain the hyper-inflammation in severe COVID-19 patients (112).
NSP6	Membrane protein potential component of the pore in DMV membranes, the pathway for coronaviral RNA products toward the cytosol (101). Interacts with Sigma-1 Receptor Sig-1R; it is suggested that Sig-1R may help in the insertion of viral replication machinery to the ER (113). Antagonizes IFN-I production. NSP6 binds to TBK1 to suppress IRF3 phosphorylation (114). Blocks STAT1 and STAT2 phosphorylation (114). Interacts with ATP6AP1, impairing lysosome activation thus blocking autophagic flux in lung epithelial cells, favoring inflammasome activation and pyroptosis (115).
NSP7	Cofactor of RdRP, together with NSP8, comprise the coronavirus polymerase complex (116). Antagonizes IFN-α signaling (117). It interacts with Ras homolog family member A (RHOA) participating in the downregulation of GTPases and the phosphorylation of vimentin and stathmin, thereby interfering with cytoeskeletal organization (118).
NSP8	Forms a complex with NSP7 that acts as an RNA primase, enhancing the RNA binding ability of NSP12 (118). Diminishes the expression of IFN-I, ISGs, and proinflammatory cytokines (119). Binds to the 7SL RNA component of the signal recognition particle, hampering protein trafficking to the cell membrane. Then it suppresses the IFN type I response and may impairs the trafficking of other proteins involved in anti-viral response (120).
NSP9	RNA binding protein phosphatase (121). Like NSP8, it binds to the 7SL RNA component of the signal recognition particle, hampering protein trafficking to the cell membrane (120). NSP9 tightly contacts with nsp12 NiRAN domain and inhibits its enzymatic activity (122). Binds to the 7SL RNA component of the signal recognition particle, hampering protein trafficking to the cell membrane. Then it suppresses the IFN type I response and may impair the trafficking of other proteins involved in the anti-viral response (120). It showed a stimulatory effect in an IFN-β-induced ISRE promoter activity (93).
NSP10	Relevant in the viral RNA capping apparatus and RNA proofreading. Co-factor is necessary for the stimulation of Nsp14 and Nsp16 (123, 124). Enhances the translation inhibitory activity of NSP14 through “structural stabilization” (125). Viral RNA modifications by NSP14 and NSP16/NSP10 complex favor evasion of immune recognition (125).
NSP11	It has an intrinsically disordered protein behavior. It is suggested to play a role in the host cytosolic membrane affinity/interaction (126).
NSP12	Along with NSP7, NSP8 makes up the complex RNA-dependent RNA polymerase (RdRp). Nidovirus RdRp-associated nucleotidyltransferase (NiRAN) domain is an enzyme that catalyzes the second step of cap structure synthesis. Catalyzes the transfer of a uridine 5′-monophosphate (UMP) to ppA to form UpppA (127). Attenuates type I IFN responses by inhibiting IRF3 nuclear translocation (128).
NSP13	It is a helicase that forms a stable complex with the holo-RdRp essential for viral replication and transcription (129). Possesses NTPase and Triphosphatase activity (130). Antagonizes distinct steps in IFN-I production. NSP13 binds to TBK1 to suppress TBK1 phosphorylation (131). Inhibits nuclear localization of IRF3 (133). Blocks STAT1 and STAT2 phosphorylation (114). Hinders nuclear factor kappa-light-chain-enhancer of activated B cells (NF-κB) phosphorylation and nuclear translocation, then it reduces NF-κB activation (131). Inhibits NLRP3 inflammasome activation in the early stage of SARS-CoV-2 infection (92).
NSP14	The ExoN domain is predicted to be necessary for maintaining replication fidelity, but it is critical for viral replication. The S-adenosyl methionin (SAM)-dependent N7-MTase is important in viral RNA 5′ capping process (132). Inhibits nuclear localization of IRF3 (133). Targets the IFN-I receptor for lysosomal degradation preventing STAT transcription factor activation (134). Inhibits translation, inhibiting the protein expression of ISGs. Reduces host protein synthesis (more than NSP1) (115).
NSP15	Coronavirus Uridylate-specific endoribonuclease (EndoU) cleaves polyuridine sequences impairing viral RNA recognition by MDA5 (135). Potently suppress primary interferon production and interferon signaling. Inhibits novo autophagy induction (136). Inhibits nuclear localization of IRF3 (133).
NSP16	S-adenosylmethionine-dependent methyltransferase (SAM-MTase) is essential for the methylation of the viral RNA cap (137). Impairs mRNA splicing, to the mRNA recognition domains of U1/U2 snRNAs, thereby it may disrupt the antiviral response (120).
Accessory Proteins	
ORF3a	Forms a non-selective cationic channel. Calcium permeability is relevant for lung homeostasis, then, ORF3a might be related to SARS-CoV-2 pathogenesis (138). Promotes lysosomal exocytosis-mediated viral release (139). Interacts with the lysosomal pathway, impairing autophagocytic activity and presumably inducing lysosomal evasion (140). Induces apoptosis in cells via the extrinsic pathway, with a weaker activity compared with SARS-CoV ORF3a (141). ORF3a primes and activate the NLRP3 inflammasome leading to the release of interleukin (IL)-1β, inflammation, and cellular death by pyroptosis (142). Activates cellular oxidative stress and proinflammatory responses producing tumor necrosis factor α (TNF-α), IL-6, and IFN-β1, probably through the activation of NF-κB (110). Inhibition of cGAS-STING mediated downstream signaling. Inhibits nuclear factor-κB signaling by blocking the nuclear accumulation of p65 (143). Mutations in this protein are probably related to immune evasion (144).
ORF3b	Suppresses induction of type I interferon (more than SARS-CoV ortholog). A longer ORF3b reading frame, related to increased antagonism was isolated from severe cases of COVID-19, suggesting it may affect viral pathogenicity (145).
ORF6	It is a potent IFN type I antagonist. Blocks IRF3 nuclear translocation through binding Karyopherin α 2 KPNA2 (146). Impairs downstream signaling of type I IFN, inhibiting ISRE and ISG56 promoters’ activation (147). Inhibits STAT1/STAT2 nuclear translocation to affect transcriptional induction of ISGs (146). ORF6 localizes at the nuclear pore complex where it binds directly to the Nup98-Rae1 complex to target the nuclear import pathway and mediate this inhibition (148). Prevents nuclear import of several host factors and inhibits the expression of transcripts. It makes infected cells, incapable of responding to invading viruses (149).
ORF7a	Inhibits IFN-I signaling. Suppresses STAT2 phosphorylation (146). Ubiquitination of ORF7a is required to block STAT2 phosphorylation (150). Binds to immune cells. Co-incubation with monocytes upregulates the expression of proinflammatory cytokines. This interaction with CD14+ monocytes impair its antigen presenting ability. It is suggested to recruit monocytes to infected lungs, favoring immunopathology of COVID-19 (151). Interferes with autophagosome acidification (134).
ORF7b	Blocks STAT1 and STAT2 phosphorylation (146).
ORF8	Highly immunogenic protein. Serological detection of anti-ORF8 IgG was suggested for acute diagnosis of COVID-19 (152). Deletions in ORF8 result in milder disease, decreased hypoxia and decreased release of inflammatory cytokines in SARS-CoV-2 infection (152). Activates Endoplasmic reticulum stress pathways (153). Favors the formation of intracellular aggregates in the lung epithelial cells (154). Targets MHC-I to lysosomal degradation. Down- I, MHC-I expression in cells impairs antigen presentation. Then CTL fail to identify SARS-CoV-2 infected cells (155). Is a type I IFN antagonist. Antagonizes IFN-β production, impairing IRF3 nuclear translocation (153). Blocks IFN-β-induced ISRE promoter activity (93). Reduces the expression ISGs (ZBP1, MX1, MX2, DHX58) in the lung epithelial cells (154). Participates in protein quality control in the endoplasmic reticulum (156). Activates IL-17 signaling pathway inducing the expression of pro-inflammatory factors favoring cytokine storm (157).
ORF9b	Targets the nuclear factor κB (NF-κB) essential modulator NEMO and interrupts its polyubiquitination, then it inhibits (IKKα)/β/γ-NF-κB signaling and IFN production (158). Locates in the mitochondria. Allosterically inhibits Htom70/Hsp90 interaction, which impairs the signaling cascade for interferon activation (159). Inhibits type I and type III IFN production. Interacts with RIG-I, MDA-5, MAVS, TRIF, STING, and TBK1, disrupt the phosphorylation and nuclear translocation of IRF3 (160).
ORF9c	SARS-CoV-2 ORF9c suppresses antiviral response. ORF9c expression impaired interferon signaling, antigen processing and presentation, complement signaling, and induced IL-6 signaling (161).
ORF10	Induces mitophagy through interaction with NIX and LC3B, leading to MAVS degradation, then it blocks antiviral signaling and favors viral replication (162).

SARS-CoV-2 SPs participate in the pathogenicity of the virus, which includes receptor recognition, replication process, viral resistance, and others (165). On the other hand, NSPs of this virus are involved in genome replication and regulation of the early stages of transcription (166). Additionally, the different SARS-CoV-2 proteins exhibit participation as virulence factors that are resistant to the immune response, among others (167–169). These biological traits of SARS-CoV-2 allow it to infect different types of cells. The virus first enters the upper respiratory tract through respiratory droplets or viral particles and then progresses to the lungs. Within the lung, the virus prefers to infect the surface of alveolar epithelial cells and cells expressing ACE2 and possessing coreceptors such as TMPRSS2 and neuropilin-1 (NRP1), among others (170). These proteins are not exclusive to the lung but are expressed on the surface of various cells in different tissues, including the ciliated epithelium, nasopharyngeal tract, upper respiratory tract, bronchial epithelium, type II pneumocytes, alveolar monocytes, macrophages, liver, kidneys, among others (171–174). This widespread expression pattern has been postulated as a potential explanation for the diverse multisystem damages observed in COVID-19 (175–178).

In the lung, the virus enters the cell through the Spike protein (Protein S), which is composed of 2 subunits (S1 and S2). The initial step of infection involves the binding of S1 to the host RBD. Subsequently, TMPRSS2 cleaves the S1/S2 region, inducing fusion of the virus with the membrane through the S2 domain (although other proteases such as endosomal cysteine proteases, cathepsin B and L, furin, basin, and neuropilin-1 are relevant, TMPRSS2 has been proved to be essential for SARS-CoV-2 pathogenesis) (179). The virus’ entry mechanism into the cell depends on multiple factors, such as cell-type physiological conditions. SARS-CoV-2 entry into the cell can be through membrane fusion or endocytic pathways (**Figure 2**) (165, 175, 180). Upon completion of viral entry, the ACE2 receptor detaches from the cell membrane, increasing serum concentrations of angiotensin II and causing vasoconstriction, inflammation, and thrombosis, as explained below in the section “Complications of organs and body systems after SARS-CoV-2 infection” (178, 180).

### 2.2 Viral progression: recognition of SARS-CoV-2 by the immune system and pathophysiology of COVID-19

After membrane fusion, the viral nucleocapsid of SARS-CoV-2 is removed, and the viral genome is released into the host cell’s cytoplasm. Viral infection generates Pathogen-Associated Molecular Patterns (PAMPS) and Damage-Associated Molecular Patterns (DAMPS), which are detected by different Pattern Recognition Receptors (PRRs) (**Figure 3**). Activation of these PRR receptors leads to activation of downstream transcription factors, such as Interferon Regulatory Factor (IRF) 3 and Kappa Light Chains of Activated B-cell Nuclear Enhancer Factor (NF-κB), which express type I and III Interferons (IFNs), along with pro-inflammatory agents, cytokines, Tumor Necrosis Factor α (TNF-α), Transforming Growth Factor β (TGF-β), Interleukin (IL)-1β, IL-6, IL-8, IL-12, IL-18 and chemokines such as Chemokine Ligand (CCL) CCL2, CCL3, CCL5, and Chemokine Ligand Motif C-X-C (CXCL) 8, CXCL9 and CXCL10 (181–183). The secreted IFNs bind to the cell surface receptor on neighboring cells to increase the expression of IFN-Stimulated Genes (ISGs) via the Signal Transducer and Activator of Transcription (STAT) 1/2/IRF9 complex, driving an antiviral state (184).

The release of these pro-inflammatory substances induces the recruitment of monocytes, neutrophils, and macrophages into the infected tissue, which in turn secrete IL-6, IL-1β, IL-8, TNF-α (181, 185), generating a concentrated medium of Reactive Oxygen Substances (ROS), which contribute to viral pathogenesis and by producing damage in the tissue surrounding the infection (185). This leads to an inflammatory feedback loop so that the more cytokines and chemokines are released, the greater the recruitment of immune cells, and with it, the greater the number of cytokines and chemokines, favoring tissue damage and increasing the severity of SARS-CoV-2 infection (186–189). As the infection progresses and innate and adaptive immune responses are intensified, highly glycosylated viral S protein with L-fucose and D-mannose-rich residues activates the complement system via the lectin pathway along with the major complement opsonin H regulatory factor (C3b) (190–192). Moreover, although complement activation is essential in the initial immune response, its sustained activity contributes to viral pathogenesis, leading to plasma clotting and tissue damage.

Thus, SARS-CoV-2 drives an exacerbated immune response, influencing the activity of natural killer (NK) cells, macrophages, and neutrophils, among other cells. So, all this immune response dysfunction continuously increases TGF-β concentrations, resulting in sustained damage to surrounding tissue and nerves in the affected area. As the damage proceeds, the cells affected by the harm release substances that further alter the affected area, compromising the permeability of the tissues and allowing the invasion of more immune cells into the tissues and organs. This sustained infiltration of immune cells, such as neutrophils, leads to the formation of Neutrophil Extracellular Traps (NETs), which increases tissue damage by forming a feedback loop (193–196).

CoVs employ diverse strategies to evade the immune response, primarily by interfering with IFN signaling, production, and resistance. The virus subsequently conceals itself within various types of cells to evade immune recognition, thus subverting the initial stages of immune defense. Furthermore, congenital defects in the host’s immune response have been linked to increased severity of COVID-19.

An early response by the host to the cytokine storm is relevant to reducing the replication and severity of SARS-CoV-2 infection, thus enabling it to promptly prepare an adaptive response. The damage generated by SARS-CoV-2 in tissues may be due to the destruction of infected cells by CD8+ T cells and the production of pro-inflammatory cytokines by other immune cells, which can produce collateral damage to tissues when fighting the viral infection (197). Some reports of this self-tolerance and cross-reactivity effect have shown that they are due to antiphospholipid antibodies, antitype I interferon, and antinuclear polymorphs, among others (198–200). This, in turn, increases the risk of contracting autoimmune diseases such as Guillain-Barré syndrome and systemic lupus erythematosus. Furthermore, according to some reports, the fact that the immune system attacks the organism simultaneously while it defends itself from the invading agent is due to the imitation or similarity of epitopes between the pathogen and parts of the organism. This could be one of the possible reasons for the severity of COVID-19 upon infections by SARS-CoV-2 (201).

Thus, considering the systemic effects of viral recognition by the immune system, long-term consequences of SARS-CoV-2 infection have been postulated to occur by viral persistence, which produces damage by viral replication products and tissue damage by chronic inflammation, where the dysregulated activation of proinflammatory pathways, lead to multiple organ system conditions (202). Some of these physiological alterations will be discussed further below.

It has been proposed that more than one of these mechanisms leads to diverse clinical manifestations. However, the greatest damage and consequences reported in Long-COVID-19 are due to indirect damage caused by the cytokine storm. In that regard, key cytokines implicated in the onset of disease severity include TNF-α, IFN-γ, IL-6, IL-1β, granulocyte-macrophage colony-stimulating factor (GM-CSF), G-CSF, and TSLP (1, 136, 203–207). To ameliorate this cytokine storm, the use of specific monoclonal antibodies for cytokines antagonistic to the JAK-STAT pathway inhibitory receptors, plasmapheresis, and the use of mesenchymal stem cells has been proposed (206, 208, 209).

### 2.3 Viral evasion and immune response to SARS-CoV-2 infection

The virus, at the beginning of the infection, hides in various types of cells to avoid immune recognition, counteracting the initial stages of immune defense, to subsequently interfere with the signaling pathways for the production and secretion of antiviral proteins, providing to SARS-CoV-2 a resistance to the IFN systems. In addition, congenital defects in the host’s immune response are associated with greater severity of COVID-19 (134, 198). The non-structural proteins Nsp1, Nsp5, Nsp13, Nsp14, and structural proteins ORF6, and ORF7 of SARS CoV-2 counteract antiviral immune response both prior to and following assembly. Moreover, as described above, SARS-CoV-2 affects the antiviral immune response by interfering with the type I IFN induction pathway and the production of other proinflammatory cytokines (**Figure 3** and **Table 2**) (133, 134, 185).

SARS-CoV-2 promotes a T helper (Th) 1 cell response and an early CD8+ T cell response, which initially helps eliminate the virus. However, in the long run, it can produce a cytokine storm (210–212). The CD4+ T cell type 1 (Th1) phenotype has been associated with promoting a state of viral control, favoring an antiviral response. In contrast, the type 2 phenotype (Th2) is associated with a greater severity of infection (213). On the other hand, circulating T follicular helper cells (cTfh) contribute to the development of neutralizing antibodies, and they have been associated with a reduction in COVID-19 severity. In addition, there are notable differences between asymptomatic and symptomatic COVID-19 patients regarding cytokine profile. Indeed, asymptomatic individuals have been shown to have a “coordinated” secretion of pro-inflammatory and regulatory cytokines, while symptomatic patients present with a polarized inflammatory reaction and a pro-inflammatory cytokine storm (214, 215).

SARS-CoV-2 has been described as using cell-to-cell transmission to spread, so the cellular response plays a role in viral control when antibody neutralization is impaired (216). On the other hand, host factors can affect the adaptive immune response. For example, it is recognized that naïve T cells are reduced in older people, which is associated with worse outcomes during infection (217). The humoral adaptive response is also relevant for controlling viral load, disease severity, and reinfection (213, 218).

When the responses of surviving and deceased patients were compared, a delay in the production of neutralizing antibodies was observed in patients with worse post-disease outcomes, which is associated with a higher viral load. The lifespan of the protective immune response produced by natural infection and vaccination is still under study and depends on multiple factors, such as the type of vaccine for the SARS-CoV-2 strain. Likewise, reports demonstrate that the immune response persists for at least 10 months up to one year upon SARS-CoV-2 infection (213, 219). Moreover, immunological memory after SARS-CoV-2 infection is observed at multiple sites, preferably in the lungs and associated lymphoid nodes (220).

In addition, studies have suggested the relationship between autoantibody levels and COVID-19 severity, so it has been proposed that self-tolerance may be affected in SARS-CoV-2 infection, leading to reactivity against host cells. Several autoantibodies have been found in patients with COVID-19, including antiphospholipid, anti- type I interferons, anti-nuclear polymorph anti-antibodies, and anti-neutrophil cytoplasmic antibodies (200, 221). In turn, some autoantibodies have been linked to a potential risk of autoimmune diseases, such as Guillain-Barré syndrome, systemic lupus erythematosus, Kawasaki disease, and autoimmune hemolytic anemia, among others (201). Although the exact mechanism underlying the development of these complications remains a topic of debate, it is hypothesized that they may result from hyperstimulation of the immune system and viral molecular mimicry. In a controlled immune response, specific T cells and neutralizing antibodies limit infection, and apoptotic cells die by phagocytosis, preventing lung damage. Conversely, a dysregulated immune response leads to an overproduction of pro-inflammatory cytokines known as cytokine storm, which directly, indirectly, or synergistically can lead to lung injury, acute respiratory distress syndrome (ARDS), and multi-organ damage in COVID-19 patients (222, 223).

In the context of SARS-CoV-2 infection, it has been postulated that T cells might have a pivotal role in the severity of clinical outcomes. Less severe disease has been observed when a prompt T cell response occurs, but this correlation is still debated (224). During natural infection by SARS-CoV-2, the whole viral particle is recognized, inducing T cell responses to several epitopes that might persist for up to 6–8 months (225). In that regard, epitope studies have identified the structural proteins ORF3, ORF8, and non-structural proteins nsp3, 4, 6, 7, 12, and 13 as dominant targets of CD4+ and CD8+ SARS-CoV-2 T cell responses (226). Likewise, T cell responses induced by vaccines have been demonstrated to have different ranges of protection. In that regard, mRNA vaccines (mRNA-1273 and BNT162b2), protein recombinant vaccine Novavax (NVX-CoV2373), or Janssen vector vaccine (Ad26.COV2.S) have been shown to induce CD4+ and CD8+ T cells that recognize a median of 11 CD4+ and 10 CD8+ SARS-CoV-2 Spike protein epitopes. Similar results have been described for inactivated vaccines, which induce T cells responses against epitopes of the Spike proteins, as well as other SARS-CoV-2 proteins included in the inactivated viral particle, both in adults and children (227–231). Particularly, the consequent T cell response against the Omicron variant persisted after 6 months: 84% for CD4+T cells and 85% for CD8+ T cells, determined by activation-induced markers (232–234). Consistently, activation of specific CD4+ T cells was detected in individuals vaccinated with Coronavac^®^ for 4 weeks after the second booster, and these cells demonstrated to be responsive against the SARS-CoV-2 Wild type and Omicron variants. Furthermore, cell-mediated immunity was shown to be more perdurable than the humoral immunity (228). Thus, considering the emergence of viral variants that evade neutralizing immunity, T-cell responses have gained importance in understanding protective immunity in SARS-CoV-2 infection (143). Observations of recovered COVID-19 patients, vaccinated and exposed individuals, support the role of memory T cells in reducing viral loads, in the prevention of SARS-CoV-2 infections and reinfections, and early presentation of multiple viral epitopes, avoiding viral escape by variants (233).

In that regard, it has been studied that the population has developed immunity through infection or vaccination after four years of spreading the SARS-CoV-2 virus worldwide. It has previously been discussed that heterogeneous immune imprinting repertoires would depend on several factors, including whether the population was vaccinated before infection, the type of vaccine, the infectious strain, and the particular conditions of the host (235, 236). Despite the above, epidemiological studies have shown that antibody levels gradually decrease over time, so they do not offer long-lasting protection against SARS-CoV-2 infection (233, 237). Moreover, considering the above, the emergence of viral variants with a repertoire of mutations in the Spike protein and the receptor binding domain (RBD) allows the virus to evade the binding and neutralization of antibodies, leading to outbreaks of reinfection around the world (237). In this context, considering the emergence of the Omicron lineage (**Table 1**), bivalent mRNA booster vaccines were developed and implemented in 2022. Booster with bivalent vaccines has demonstrated greater vaccine efficacy than monovalent vaccines against the Omicron subvariants in all age groups, especially against severe clinical outcomes (238, 239). A systematic evidence synthesis and meta-analysis, conducted up to December 2022, summarize the evidence regarding the long-term effectiveness of COVID-19 vaccines in preventing infections, hospitalizations, and mortality (240). The WHO defined adequate levels of protection of vaccines against COVID-19 for infection. In the first series of primary pandemic vaccines, protection against infection was 83% and 62% after 42 days and 139 days post-immunization, respectively. This study revealed that the baseline levels of effectiveness of the vaccine against the Omicron variant were 70% against infection, and these decreased to 43%. Of note, protection levels against hospitalization also decreased from 89% at baseline to 71%, and there was insufficient evidence on effectiveness against Omicron wave mortality (240). Therefore, it is necessary to address new COVID-19 vaccination strategies to improve protection levels among the population.

## 3 Organ and body system complications upon SARS-CoV-2 infection

As mentioned above, SARS-CoV-2 mainly infects the lungs but can also affect other organ systems. SARS-CoV-2 infection to various host organs has been proposed due to the heterogeneous distribution of ACE2 and TMPRSS2. In addition, the damage caused by SARS-CoV-2 infection and the post-infection sequelae produced by COVID-19 can be highlighted, which includes lung, kidney, and liver damage, among others (241–243). The most common sequelae and complications of severe COVID-19 are fatigue, dyspnea, cognitive impairment, anxiety, depression, anosmia, and ageusia (243). In contrast, in mild or asymptomatic COVID-19, fatigue, myalgia, headache, cough, and mucus are present (**Table 3**) (243).

**Table 3:** Pathophysiological manifestations, clinical signs, and clinical presentation and/or sequelae of SARS-CoV-2 infection in different systems.

System	Manifestations			References
	Physiopathological	Signs	Clinical presentation	
**Pulmonary**	• Decreased tissue repair. • Proliferation of myofibroblasts and alveolar remodeling. • Lung injury induced by prolonged mechanical ventilation (VILI) in Pulmonary Fibrosis (FP). • Collagen deposit in the lungs. • Infiltration of inflammatory cells.	• Pulmonary fibrosis (PE). • Pneumonia. • Thromboembolism. • Frosted glass opacity (GGO). • Sepsis due to COVID-19. • Trauma from prolonged mechanical ventilation (VM). • Thromboembolism. • Dysregulation of pulmonary immune response. • Microvascular thrombosis. • Alveolar damage. • Respiratory failure.	• Decreased lung capacity. • Pleural effusion (PE). • Oedema. • Hemorrhage. • Hypoxia.	(244–249)
**Cardiac**	• Acute, myocarditis. • Heart failure. • Endothelial lesions.	• Hypertension. • Thrombosis.	• Tachycardia. • Arrhythmias. • Atrial fibrillation.	(1)
**Renal**	• Nephropathy. • Infrarenal inflammation. • Increased vascular permeability. • Volume depletion.	• Damage to tubular cells. • Congestion of the renal veins. • Renal hypotension. • Renal hypoperfusion.	• Kidney damage. • Cardiorenal syndrome type 1.	(250–253)
**Muscular**	• Myopathy. • Myalgia. • Myositis. • Electrolyte anomalies.	• Rhabdomyolysis.	• Fatigue.	(253, 254)
**Hepatic**	• Abnormal increase in aspartate aminotransferase (AST) and alanine aminotransferase (ALT). • High levels of bilirubin. • Lobular necroinflammation. • Sinusoidal microthrombi.	• Jaundice. • Coagulopathy. • Hepatic encephalopathy.	• Liver damage. • Macrovesicular steatosis. • Hepatitis. • Convulsion.	(253, 255–257)
**Nervous**	• Meningitis. • Encephalitis. • Polyneuropathy. • Transverse myelitis.	• Acute necrotizing hemorrhagic encephalopathy. • Guillain-Barré syndrome.	• Myalgia. • Headache. • Confusion. • Lethargy. • Disorientation. • Agitation. • Sleepiness. • Seizures.	(258–261)
**Oral and nasal**	• Edema located in the olfactory cleft. • Salivary gland infection. • Damage to the olfactory epithelium.	• Partial or total invasion or destruction of the nasal and/or oral epithelium.	• Anosmia. • Hypogeusia. • Hyposmia. • Ageusia. • Cacosmia. • Phantosmia. • Rhinorrhea. • Nasal congestion.	(262–267)
**Gastrointestinal**	• Cellular damage of the gastrointestinal system. • Dysbiosis.	• Diarrhea. • Vomiting. • Nausea.	• Abdominal pain. • Gastroenteritis. • Cramps.	(268, 269)
**Immune**	• Elevation of TNFα, IL-6, IL10, IL-1β, IL-2, IL-8, IL-17, G-CSF, GM-CSF, IP10, MCP1 and MIP1α2. • T-cell apoptosis. • Deficiency of natural killer (NK) cells. • Release of extracellular neutrophil traps (NETs).	• Immunosuppression. • Abnormal maladaptive immune response. • Cytokine storm.	• Fever. • Immunosuppression. • Lymphopenia. • Neutrophilia or neutropenia.	(269, 270)
**Circulatory**	• Increased D-dimer. • Increased fibrinogen.	• Hypercoagulation. • Bacteremia.	• Intravascular thrombosis. • Ischemic strokes. • Cardio embolism.	(271, 272)
**Ocular**	• Epiphora • Enlargement of the ocular arteries and veins. • Infection of the ocular epithelium.	• Conjunctival hyperemia. • Chemosis.	• Conjunctivitis. • Blurred vision.	(273, 274)
**Pancreatic**	• Pancreatic lesion.	• Pancreatitis. • Hyperglycaemia.		(275, 276)
**Reproductive system**		• Devastation of the testicular parenchyma.	• Testicular discomfort. • Infertility. • Menstrual disorders. • Dysregulation of the menstrual cycle. • Preterm birth. • Preeclampsia. • Gestational hypertension.	(277, 278)

### 3.1 Renal complications

The kidneys express a high concentration of ACE2, TMPRSS2, and furins. At the same time, the kidneys play an important role in controlling blood pressure through different mechanisms, such as the Renin Angiotensin Aldosterone System (RAAS). SARS-CoV-2 can be distributed throughout the body through the blood, infecting organs and tissues susceptible to the presence or absence of the receptors necessary for viral entry (279–281). Kidney damage is not only produced by the direct action of the virus by destroying tissues, but it can also result from an overactivation of the immune system (281).

SARS-CoV-2 damages renal tubules by inducing the infiltration of macrophages and complement proteins such as C3a and C5b-9, leading to tissue fibrosis (282). This damage causes waste to accumulate in the kidney, producing different multisystem effects. Additionally, damage to the kidney generates an imbalance of the ACE 2/Ang 1–7/MasR axis, which leads to an imbalance of the RAAS (281, 282). If kidney damage occurs together with lung damage, hypercapnic acidosis occurs, which contributes to aggravating multisystem damage. By lowering the price and not being able to activate the RAAS adequately, it causes kidney and heart damage, generating a hypovolemic state, which in turn produces vasoconstriction, leading to a procoagulant state, injuring small-caliber arteries and ducts (282–284).

The cumulative damage caused by SARS-CoV-2 results in toxins and organic waste accumulation. As it destroys tissues, the waste products enter the bloodstream, accumulating in the kidneys already compromised by the viral infection and the hyperactivated immune system. This exacerbates a detrimental loop, reaching a critical juncture where the abundance of toxins and waste in the organism compromises additional tissues, ultimately culminating in renal and/or hepatic failure (285–287).

### 3.2 Cardiovascular complications

When SARS-CoV-2 reaches the heart, it causes heart damage, myocardial injury, arrhythmias, and heart failure. This damage to the heart can be generated directly by the virus or by the exacerbated immune response. As SARS-CoV-2 enters and destroys cells, their functions are altered, leading to cardiac arrhythmias, deep vein thrombosis, pulmonary embolisms, and decreased lung volume (288). People with heart disease before infection tend to have more severe symptoms or develop complications during and after COVID-19, including the development of hypoxemia, coagulopathies, cardiac arrest, and damage (289). During infection, cardiac cells release troponin, and troponin concentrations have been reported to correlate with disease severity and can be considered a biomarker (284, 290–293). Synergistically, due to cardiac damage, a decrease in blood pressure occurs, leading to the activation of the RAAS system, which in turn triggers vasoconstriction, vascular inflammation, and an increase in Angiotensin 2 levels, contributing to the pathogenesis and cardiovascular complications of the infected individual. In addition, it has been shown that SARS-CoV-2 causes coagulation disorders, producing hyper coagulopathies and forming thrombi, which can trigger embolisms in different organs, causing the death of tissues or cells that release toxins and subsequently travel through the blood. These cellular wastes accumulate in the kidneys, which will hinder their functions. Likewise, the released toxins accumulate in the blood, potentially causing peritonitis (294–297).

### 3.3 Metabolic complications

SARS-CoV-2 can induce widespread metabolic effects, including insulin resistance, dyslipidemia, diabetes, and systemic inflammation. In particular, alterations in the pancreas and liver of individuals infected by SARS-CoV-2 can lead to a state of hyperglycemia due to metabolic stress, leading to the development of “New Onset Diabetes (NOD). This condition can significantly impact the course of the infection. However, the precise mechanisms underlying the emergence of these complications and new pathologies have not been fully elucidated (298–300).

One explanation for this is that SARS-CoV-2 infection in adipose cells can cause a decrease in insulin sensitivity, which can lead to hyperglycemia, leading to an increase in lipids in the body, which can lead to dyslipidemia (301), this affects the pancreas and liver, causing a deterioration in the function of these organs. Furthermore, since SARS-CoV-2 can infect the CNS and the Peripheral Nervous System (PNS), it has been reported that damage to either of these two systems, either at the nervous level or at the functional level, produces a cascade effect altering normal metabolism (302, 303).

### 3.4 Pulmonary complications

Pulmonary complications are prevalent conditions during and after SARS-CoV-2 infection. Common complications include pulmonary fibrosis, respiratory failure, pulmonary embolism, and secondary bacterial pneumonia. The damage to the pulmonary epithelium is due to either a direct action of the virus or the effects of the immune system in a small space. This damage and effects lead to pulmonary fibrosis, making breathing difficult. Moreover, immune cells are recruited, which release TNF-α, IL-1β, IFN-γ, and GM-CSF (304, 305). Additionally, as immune cell recruitment occurs at the site of injury, these cells also secrete Proteolytic Enzymes such as Matrix Metalloproteinase (MMP) that degrade the extracellular matrix, producing the release of cytokines, TGF-β and Chemoattractant Protein (MCP) 1, which in turn induces the detachment of fibroblasts, collagen, and cellular matrix components, aggravating pulmonary fibrosis (268, 306–308).

### 3.5 Gastrointestinal complications

COVID-19 has been reported to produce gastrointestinal complications due to damage to gut epithelial cells, which activates the innate immune response. Indeed, activated dendritic cells, macrophages, and NK cells target the site of infection and secrete IL-1β, IL-6, and TNF-α, while activated CD4+ T and CD8+ T cells produce IFN-γ among other inflammatory cytokines that aggravate tissue damage (309, 310). As damage increases in the intestine, cell death, cellular alterations, and pathological complications increase, hindering the absorption of nutrients, increasing intestinal permeability that induces a greater filtration of immune cells, repeating the cycle and damaging the gastrointestinal mucosal barriers. This, in turn, leads to less water reabsorption in the kidneys and intestines, which could explain the persistent diarrhea and increased nutritional disorders that occur in COVID-19 patients. Moreover, intestinal damage impairs gastrointestinal motility, fluid, and electrolyte secretion, contributing to nausea, vomiting, and abdominal discomfort (311, 312). Gastrointestinal damage drives macrophages and T lymphocytes to the injury site, secreting IL-1β, IL-6, IL-8, IFN-γ and CCL2, CCL5, and CXCL8, which can act synergistically to recruit more inflammatory cells, thus aggravating intestinal inflammation (313–315).

### 3.6 Hematological complications

The most common hematologic complications caused by SARS-CoV-2 are thrombocytopenia, thrombosis, disseminated intravascular coagulation (DIC), and platelet dysfunction. While these disorders have been seen in repeated cases of COVID-19, the underlying causes of these abnormalities have not been fully elucidated. Some hypotheses suggest that these effects result from the destruction of platelets in the circulation due to an antibody cross-reaction resulting from mimicry of viral epitopes with those of platelets. This hypothesis is based on the fact that the antibodies generated to fight the virus attack the platelets by mistake, or the latter is in the “crossfire” of the immune system against the virus (316–318).

On the other hand, other hypotheses suggest that this effect is because the virus could infect platelet precursor cells in the bone marrow, which would alter their function (317). Furthermore, it is known that proinflammatory cytokines such as TNF-α, IL-1β, and IL-6 can alter the coagulation factor system by increasing the production of fibrinogen, Von Willebrand Factor (vWF), Factor VIII, and Factor XII, which increase the risk of coagulopathies (319–321).

### 3.7 Neurological complications

It has been postulated that SARS-CoV-2 could infect CNS cells, which explains the cognitive and sensory symptoms patients experience. It has also been proposed that the virus could cross the blood-brain barrier, affecting the recognition of signals traveling from the PNS to the CNS (302). Alternatively, based on cerebral Positron Emission Tomography (PET), it has been proposed that patients with prolonged COVID-19 who change their brain metabolism have a functional alteration of cells, which do not process or transmit information properly. This effect is greater in the bilateral brainstem, which causes hyposmia, anosmia, memory impairment, cognitive impairment, and pain (303). Other hypotheses have proposed that the virus, by damaging nerves and receptors for olfactory and gustatory signals, causes ageusia and anosmia. This damage to the receptors could be due to the direct action of the virus or collateral damage from the overreactive immune system in the nervous tissue environment (322–325). Other complications observed in the brain are encephalopathy, confusion, dizziness and alterations in memory or perceptions, Cerebrovascular Accidents (CVA), transverse myelitis, memory loss, decreased reflexes, neuropathies, myopathies or Guillain-Barré syndrome (322–325).

### 3.8 Post COVID-19 condition

It has been described that the risk of long-term sequelae after the resolution of COVID-19 is proportional to the age and health status of the patient. Similarly, patients with a more severe infection during COVID-19 tended to have a higher probability of developing sequelae of varying severity and durability (326–328). The post-COVID-19 sequelae can be generated by various factors, such as the fact that SARS-CoV-2 uses some cells as a reservoir, entering a latency period and affecting cell function while waiting for regrowth or activation (183, 328). Other theories suggest that these sequelae are generated by damage to nerves and cells due to a direct action of the virus on nerve cells and receptors or by indirect damage produced by an exacerbated immune system that, in its function of counteracting the infection, can cause collateral diseases, affecting nerve cells and receptors (1, 183). The most common complications are chronic fatigue syndrome, sleep disorders, breathing problems or asthma, heart problems, neurological or psychological problems, anosmia, and ageusia (329, 330). Finally, other theories point out that due to SARS-CoV2 infection, the cellular and organic metabolism is altered. Specifically, the body and systems try to readapt to these new conditions, and it is during this readjustment that some functions are partially or permanently changed, thus generating a loss of tastes, sensations, and confusion of tastes or smells (330–332). In the latter hypothesis, the receptors and nerve cells remain intact. However, they can no longer process information as before SARS-CoV-2 infection (333, 334).

It is known that, in order of frequency, the symptoms of COVID-19 are fever (98%), cough (82%), and, in a lower percentage of cases, difficulty breathing (to be determined). On the contrary, the severity and symptoms of Long-COVID-19 are diverse, and their frequency is highly variable. Particularly, it is challenging to elucidate the frequency of long-term COVID-19 symptoms. However, it has been proposed, based on the number of cases, that the frequency of these, from highest to lowest, would-be cardiovascular conditions, thrombotic conditions, cerebrovascular conditions, diabetes, encephalomyelitis, dysautonomia and metabolic alterations, myalgic encephalomyelitis (ME) and chronic fatigue syndrome (CFS), which is the least common (335–341). It is important to note that ME and CFS, along with postural orthostatic tachycardia syndrome (POTS), can be lifelong (342). It has been reported that between 2 to 14% of patients who had COVID-19 present with conditions similar to or compatible with POTS during Long-COVID-19. These conditions include tachycardia, orthostatic intolerance, fatigue, cognitive and muscular impairment from 6 to 10 months (343, 344). Moreover, it has been postulated that these symptoms develop due to autoimmune disorders, autonomic dysfunction, direct damage from SARS-CoV-2 infection to nerve cells, and invasion of the virus into the CNS (343–346). At the same time, due to the different mechanisms that SARS-CoV-2 has to induce damage in the body, as well as affect the bioavailability of ACE2 and its presence in the various organ systems, other conditions can develop, such as metabolic diseases and those that affect the thyroid, kidney, and liver (347).

In addition, it has been reported that Graves’ disease and Hashimoto’s thyroiditis are among the thyroid conditions of long-term COVID-19. These conditions are thought to be induced by the cytokine storm during COVID-19 and by the molecular mimicry of components resulting from the viral infection with those of the host (347–351). Other conditions that have been reported are lymphopenia, neutropenia, and oxidative damage. However, these sequelae do not have a high frequency, so they have not been directly attributed to a Long-COVID-19 pathology but rather to late COVID-19 symptomatology due to SARS-CoV-2 viremia disseminated (352, 353).

Despite the consequences of Long-COVID-19, there is not enough information to estimate the frequency and severity of each pathology present in this disease. To overcome these limitations, studies with a larger number of patients are needed to assess the frequency, and exhaustive studies of each clinical manifestation are required to estimate severity.

### 3.9 Viral factors

The susceptibility to SARS-CoV-2 infection and the severity of COVID-19 are contingent upon various variables and diverse factors pertaining to both the host and the virus. Essential factors encompass viral load, replication rate, immune evasion capacity, and the pathogen’s virulence. Concurrently, host-related factors, including age, sex, immune status, comorbidities, smoking habits, and genetics, among others, play crucial roles in determining the infection’s outcome and the disease’s progression.

At the beginning of the pandemic, SARS-CoV-2 had high fidelity in the replication of its genome, but as it spread, it interacted with many other hosts, which caused this virus to mutate and generate new high-impact variants (354–358). In this sense, the reproduction number (R0) value for the first variants was ~2.9, but as it spread and generated new variants, the R0 increased, becoming more significant than 7.0 in the later variants.

SARS-CoV-2 variants tend to have different distinctive features, such as better affinity with ACE2, more resistance to the environment, and greater ability to evade the immune response, among other effects. There is still the possibility of mutations that alter the course of infection, leading to lower efficacy of current vaccines and prophylactic measures, which may ultimately lead to a new variant not affected by current vaccines (**Table 1**) (359–361). It has been described that the Alpha variant contains mutations in sub-genomic RNA, improving efficiency and immune evasion. Other mutations, such as those associated with ORF 3a, ORF 6, and ORF 7a, provide a better ability to evade the immune response, highlighting the need to keep this viral agent under continuous investigation (362, 363).

Although SARS-CoV-2 encodes an RdRp complex and additional non-structural protein complexes that provide corrective activities, increasing the fidelity of viral replication, this fidelity does not always fulfill its corrective function by producing mutations in the genome and generating new variants with different capabilities (354–358). In the Delta and Omicron variants, the R0 was ~2.79 and ~7.0, respectively, suggesting that each variant’s mutation rate is increasing (357, 364). Other factors that modulate circulation and the appearance of variants include immunocompromised people and the implementation of COVID-19 vaccination.

## 4 Host-related risk factors

According to the literature, commonly reported risk factors in severe cases of COVID-19 include comorbidities such as kidney injury, chronic obstructive pulmonary disease (COPD), hypertension, diabetes, cardiovascular diseases, cancer, and obesity. Also, the gender and age of the patients are considered risk factors (365, 366).

### 4.1 Gender

It is known that there are immunological differences between men and women in susceptibility to certain infections due to, among other factors, anatomic characteristics of each gender (such as the width of the respiratory tract), metabolism, etc. (366). Although SARS-CoV-2 has been reported to affect men and women equally, more severe cases of COVID-19 occur in men more frequently compared to women, possibly due to the anatomy and physiology of each gender (367). Genetics may also be involved in the pathological differences and their respective severities. It is known that the X chromosome encodes genes related to innate and adaptive immune responses, since the genes for chemokine receptors (CXCR) 3, Toll-like receptors (TLR) 7 and CD40 ligand (CD40L) are expressed in it (368, 369). On the other hand, unlike women, a generalized inflammatory response can occur in men, probably due to immune plasticity associated with patterns of expression of genes encoded on the X chromosome (370, 371). These differences are also reflected at the level of hormones, where androgens, mostly produced in men, can increase the expression of ACE2 and TMPRSS2 in lung epithelial cells. Moreover, testosterone has been shown to decrease the immune response (370–372). On the contrary, estrogen, considered one of the female sex hormones, has anti-inflammatory properties that lead to a higher degree of immune modulation and induces a downregulation of ACE2 expression on type 2 pneumocytes, endothelial cells and kidney cells, among others (372–375). Immunological differences between men and women have been shown to affect susceptibility to infectious diseases, the immune response produced against pathogens and the effectiveness that certain vaccines can provide (366–369). In conclusion, the role of gender in the severity of COVID-19 is still unclear, so it is essential to continue studying it.

### 4.2 Obesity

It is important to consider that the underlying characteristics of individuals will dictate the physiological response to infection. Clinical evidence has shown that obesity is a factor to consider when defining the susceptibility and severity of COVID-19. Specifically, there is a Body Mass Index (BMI) scale, in which individuals with: a BMI of 18.5 to 24.9 kg/m2 are not obese; a BMI of 25 to 29.9 kg/m2 are overweight; a BMI of 30 to 34.9 kg/m2 have a type I obesity; a BMI of 35 to 34.9 kg/m2 have a type II obesity; and a BMI ≥ 40 kg/m2 have a grade III obesity. Likewise, degrees of obesity have been related to a less favorable prognosis during COVID-19 compared to those individuals with a BMI less than 29.9 kg/m2. Moreover, patients with a BMI ≥ 30 kg/m2 have shown greater admission rates to the Intensive Care Unit (ICU) compared to those with a lower BMI (376, 377).

Indeed, a high BMI has been correlated with complications such as increased respiratory symptoms, kidney damage, coagulopathies, thromboembolisms, and increased mortality in patients with COVID-19. Furthermore, obesity is commonly associated with metabolic alterations such as insulin resistance, alteration of serum glucose levels, dyslipidemia, etc. These health alterations, combined with the stress caused by the SARS-CoV-2 infection itself, can lead to a worsening of comorbidities or the appearance of new pathologies, such as NOD (376–378).

Furthermore, obesity is also characterized by a dysfunction of the immune system. In fact, it has been reported that patients with a high BMI have been correlated with a higher frequency of the anti-inflammatory CD4+ T cell subsets Th2 and regulatory T cells that can make it difficult to resolve a viral infection, such as that caused by SARS-CoV-2 (376, 378). Moreover, SARS-CoV-2-specific IgG antibodies have been reported to be negatively associated with BMI in COVID-19 patients. Likewise, there has also been shown a positive correlation between serum C-reactive protein (CRP), serum amyloid protein, and ferritin levels with BMI, which could be used as predictive biomarkers for dysfunctional immunity and severity of COVID-19 (378, 379). Besides that, the excess of adipose tissue has been defined as a chronic state of inflammation or “low-grade inflammation”, where proinflammatory cells (macrophages, dendritic cells, cytotoxic T cells, and Th1 cells) accumulate in it, particularly in visceral fat. Therefore, this pro-inflammatory environment in patients with higher BMI favors the amplification of the COVID-19 cytokine storm and, in turn, the exacerbation of the severity of the disease (379–381). Specifically, secretion of IL-6 and high levels of leptin, TNF-α, CXCL-10 have been observed in obese patients with a severe COVID-19 disease.

In addition, ACE-2 is highly expressed in visceral fat, suggesting an important role of adipose tissue in the severity of COVID-19. However, studies on the effect of overexpression of ACE2 in this type of tissue are still inconclusive (381). Nevertheless, it has been suggested that due to ACE-2 expression, adipocytes act as a reservoir of SARS-CoV-2, making more difficult viral clearance.

Furthermore, adipocytes can secrete TNF-α, CXCL10 and leptin. The latter is an immunoregulatory protein since several immune cells express the receptor for this protein. Leptin, when is activated, produces the induction of different downstream signaling pathways such as: the Janus Kinase (JAK) signal transducer and the STAT pathway; the phosphatidylinositol-3-kinase (PI3K) pathway; and the Mitogen-Activated Kinase (MAPK) pathway. As a result, an inflammatory phenotype is induced, including the secretion of IL-6, IL-12, and TNF-α by monocytes and ROS production by neutrophils, which stimulates activators of CD4+ T and CD8+ T cells. Additionally, adiponectin decreases in obesity, favoring a dysregulation of endothelial homeostasis and an exaggerated inflammatory response. On the other hand, weight gain could lead to leptin resistance, which, in turn, increases the frequency of Treg cells in tissues, leading to an immunosuppressive phenotype. Consequently, the effective elimination of SARS-CoV-2 infection is affected, increasing the severity of the disease (382, 383). Therefore, adipokine dynamics in obese individuals contribute to an immunological imbalance, which is relevant to the outcome of COVID-19 patients with this condition. Taken together, ACE2 overexpression in visceral fat, metabolic alterations, and immune imbalance may contribute to a poor prognosis during and after SARS-CoV-2 infection in obese COVID-19 patients (377, 383, 384).

### 4.3 Age

Some reports suggest that the severity of COVID-19 varies with age, with older adults facing an increased risk and severity of the disease, as well as a higher likelihood of experiencing sequelae and death compared to children and young adults. However, it is worth noting that the impact of COVID-19 on children remains a topic of debate and ongoing research (377, 384, 385). It has been postulated that the variation in the severity of COVID-19, between different age groups, could be attributed to immune immaturity in children and immune senescence in older adults (386, 387). Interestingly, although both age groups receive similar viral loads after SARS-CoV-2 infection, children tend to be asymptomatic or present mild symptoms (unless they develop a pathology known as Chronic Multisystem Inflammatory Syndrome, which is associated with unfavorable outcomes in children under 5 years of age) (388). In patients under 20 years of age, increased levels of CXCL10 and GM-CSF, together with T and B cells in the blood, produce a hyperreactive immune response, causing damage to different tissues (386).

### 4.4 Diabetes

It has been reported that high blood glucose levels impair the immune response. Therefore, diabetes favors infectious diseases, which are directly related to an increase in the severity and mortality of COVID-19 patients (389). In fact, patients with abnormal blood glucose levels tend to have coagulation imbalance, endothelial dysfunction, and alveolar and extrapulmonary macroangiopathy (390–392). In patients with type 2 diabetes, neutrophils, macrophages, monocytes and NK cells have altered function, decreasing the release of cytokines and IFN-α necessary to eliminate viral infections (393–396). Furthermore, metabolic alteration of glucose and lipids leads to a pro-inflammatory state by altering the synthesis of pro-inflammatory cytokines of immune cells (396, 397). Particularly, it has been postulated that diabetes affects antibodies at the glycosylation level of immunoglobulins, which increases the action of the Major Histocompatibility Complex (MHC) Class I on myeloid cells, contributing to the development of a hyperreactive immune response (24, 398, 399). Although the evidence on whether glucose levels increase susceptibility to SARS-CoV-2 infection is inconclusive, greater severity of COVID-19 has been reported in hyperglycemic patients (390). A possibility for this increase in disease severity may be that these patients have hypercoagulopathies, leukocytosis, and neutrophilia, among others (25, 26). In addition, patients with hyperglycemia increase the expression of ACE2 in the pulmonary, renal, and cardiac systems (391, 392). Of particular interest is NOD, which, as mentioned above, has been associated with SARS-CoV-2. Although the exact mechanisms by which it develops are not entirely clear, the main theories point to undiagnosed (latent) diabetes that worsens after COVID-19. Other theories point to hyperglycemia due to metabolic stress and damage to the β cells of the pancreas due to the direct action of the virus or the indirect action of the immune system (298, 392). While scientific evidence is inconclusive on susceptibility to SARS-CoV-2 infection in diabetic patients, there is a correlation between the severity of COVID-19 disease and blood glucose and insulin levels (27, 400). In addition, insulin affects blood pressure, which in turn causes activation of the RAAS, further increasing this multisystem effect and ACE2 concentrations in the body.

### 4.5 Nephropathy

Patients with kidney problems have been observed to present significant clinical manifestations during COVID-19, often resulting in complications such as acute kidney injury (AKI). This nephrological complication is characterized by altered values of the estimated Glomerular Filtration Rate (eGFR), serum creatinine, proteinuria, hematuria, and abnormal blood urea (BUN) levels. In particular, people with AKI are more likely to require ICU admission and tend to experience less favorable outcomes after SARS-CoV-2 infection (28, 392). One hypothesis explaining this phenomenon suggests that as the virus infects various cells and tissues, subsequent cell death releases waste materials, further contributing to the overactive immune response. These waste products and substances reach the kidney, causing this organ to function at an accelerated rate. Likewise, since the virus can still infect kidney cells and compromise their normal function, the organ becomes stressed and eventually develops AKI (28, 30, 401). Clinical management is crucial to mitigate the severity of kidney injury, which requires careful monitoring of kidney function in patients with COVID-19. Reports indicate patients may continue to shed viral loads for prolonged periods after COVID-19. This persistence could result from a dysfunctional immune response during SARS-CoV-2 infection. Alternatively, the virus may remain dormant in the system or adopt a latent form in airway epithelial cells, which could explain the epithelial damage and rearrangement observed in the post-COVID-19 period. Another possibility is that the virus persists in the cells of the nervous system, which could explain the consequences and damage observed after SARS-CoV-2 infection in sensory functions of the body, such as taste or smell (31, 32). Patients with acute renal failure (ARF) have also been reported to have a higher rate of SARS-CoV-2 infection than those without ARF. In addition, patients undergoing ARF, or chronic kidney disease (CKD) are reported to have a higher frequency of ICU admission and death (33, 36).

### 4.6 Cardiovascular disease

SARS-CoV-2 has the potential to directly and indirectly affect the cardiovascular system. On the one hand, direct effects include the destruction of cells of the cardiovascular system that express ACE2 and TMPRRS2. On the other hand, the virus indirectly affects the circulatory system. As the infection of SARS-CoV-2 progresses, there is an increased risk of cardiovascular complications, such as elevated coagulopathies due to alterations in the coagulation cascade and an elevated likelihood of inflammation in various tissues, particularly in the endothelial and vascular systems. This heightened inflammation contributes to cardiovascular events, including myocardial infarction, venous thrombosis, and coronary damage. Individuals with pre-existing cardiovascular conditions during COVID-19 have been reported to exhibit altered levels of biomarkers such as C-reactive protein (CRP), D-dimer (DD), tissue factor (TF), and Von Willebrand factor (VWF). These changes in biomarker levels indicate the potential impact of SARS-CoV-2 on the cardiovascular system, emphasizing the need for comprehensive monitoring and treatment in individuals with cardiovascular conditions (34, 402, 403).

It has been shown that people with pre-existing conditions such as coronary heart disease, high blood pressure, history of cardiovascular disease, or coronary heart disease are more likely to develop post-COVID-19 sequelae (43–45). As previously discussed, in the renal section, cell death results in the release of potassium into the bloodstream. Under normal circumstances, the kidneys filter potassium. However, when the kidneys are overloaded, their functioning is impaired. The increased potassium levels in the blood activate voltage-gated potassium channels (KV), inducing an altered electrical response in muscle fibers. This alteration affects blood pressure by causing a decrease in adenosine triphosphate (ATP), subsequently activating ATP-dependent potassium channels (KATP). Activation of KATP leads to a reduction in heart muscle contractions, which contributes to a decrease in blood pressure. This decrease in blood pressure activates the RAAS system, causing the synthesis of ACE2. When SARS-CoV-2 induces the activation of the RAAS system, it triggers the depletion of ACE2 on the cell surface, increasing angiotensin I in the blood, which could contribute to the development of atherosclerosis (43–45).

Atherosclerosis can manifest itself through several mechanisms: a) First, ACE2, by converting angiotensin I to angiotensin II, helps raise blood pressure. However, if there are low levels of ACE2, angiotensin II is not produced, consequently angiotensin I levels will increase; b) Secondly, ACE2 is an enzyme that facilitates the removal of cholesterol from the arteries. In the absence or reduction of ACE2, cholesterol can accumulate in the arteries, leading to plaque formation; and c) Third, ACE2 functions as an enzyme that plays a protective role for endothelial cells, which line the inside of arteries. Therefore, when ACE2 is deficient or decreased, endothelial cells may become more vulnerable to injury, contributing to plaque formation. The reduction in ACE2 expression induced by the virus may thus contribute to the onset of atherosclerosis through multiple pathways, further complicating the symptomatology of COVID-19 (34, 35, 46).

### 4.7 Immunocompromised conditions

Immunocompromised individuals who may have this condition due to a genetic disorder or having an underlying pathology, such as one caused by a pathogen or having received a transplant, constitute a group of particular interest. This is attributed to the alteration observed in immune responses within this population, which subsequently influences the clinical course of various pathologies.

a) Immune disorders due to congenital problems: More than 95 inborn errors of immunity, also called primary immunodeficiency syndromes, have been reported (47). These genetic disorders are a group of conditions that impact the immune system from birth. These disorders compromise the immune system’s ability to effectively fight pathogens, making people more susceptible to recurrent infections and, in some cases, ending up with autoimmune conditions. In the context of COVID-19, people with these genetic disorders have been reported to experience prolonged durations of SARS-CoV-2 infection. Prolonged virus shedding times after infection cause a higher likelihood of severe illness, less favorable prognoses for resolution of infection, and a higher risk of developing serious complications after COVID-19, including pneumonia and sepsis, among others (49, 50, 404).
b) Immune disorders due to infectious agents: Certain infectious agents, such as the human immunodeficiency virus (HIV) or bacteria such as *Streptococcus pneumoniae*, can weaken the immune system, either by suppressing or altering the body’s immune response. Theoretically, it has been postulated that individuals in this group may experience greater severity of COVID-19, due to the combined impact of the damage caused by their existing pathogens, the effects of the virus itself, and the compromised immune system. Additionally, other opportunistic infectious agents may take advantage of this depressed immune state. This complex condition poses challenges for healthcare, as treatments must target not only the SARS-CoV-2 infection, but also the coexisting pathogens that contribute to comorbidities in these patients (49–52, 404).
c) Immunological disorders due to immunosuppressants: Patients who undergo organ or cell transplants, such as hematopoietic cells (HTC), or solid transplants (SOT), have a higher rate of admission to the ICU and a greater severity of COVID-19 (50). It is thought that in these patients, SARS-CoV-2 has a longer viral replication time and, therefore, a greater viral load is produced, leading to more significant and sustained pathology (49). However, the results of this type of research that have been carried out so far remain ambiguous. For example, other studies have reported low rates of SARS-CoV-2 infection among transplant patients. Although, this can be attributed to the strict health measures implemented for these patients (53).

Therefore, more extensive investigations are required to elucidate the mechanisms behind the pathogenesis of SARS-CoV-2 infection in immunocompromised patients.

### 4.8 Microbiota

It is well known that several microorganisms live in a symbiotic relationship in the human body, called Microbiota. These microorganisms produce molecules and vitamins, such as vitamin K, which are used by the human body and, in turn, provide nutrients and protection to these microorganisms (54). The Microbiota is characteristic and specific to everyone. This microbiota aids digestion and protects the host from possible pathogenic microorganisms (55). It has been reported that the microbiota has immunoregulatory functions. In fact, changes in this microbiota induce a dysbiosis that affects the host by exposing it to opportunistic pathogens, or triggering altered immune responses such as allergies or inflammatory bowel disease (IBD), which increases the probability of secondary diseases, such as obesity and diabetes (54–56).

During SARS-CoV-2 infection, bacterial co-infections or dysbiosis may occur, contributing to increased mortality and morbidity in individuals. This effect has been previously reported in CoVs such as SARS-CoV and MERS-CoV, with co-infections of each of the respective viruses together with respiratory tract pathogenic bacteria such as *Streptococcus pneumoniae* and *Staphylococcus aureus* (57). Concentrations and types of microbial populations vary upon a viral infection. Specifically, it has been shown that 3 days after SARS-CoV-2 infection, *Fusobacterium periodonticum* in the oral cavity decreased significantly (58). It has also been reported that *Faecalibacterium prausnitzii*, a vital bacterium involved in anti-inflammatory processes, is negatively altered as the severity of COVID-19 increases (58, 59). Additionally, a reduction in host-typical commensal bacteria, along with increased presence of pathogens, has been correlated with increased severity of COVID-19. This association may be related to the role of commensal bacteria in shaping the immune system response, influencing the outcome of viral infections. However, the specific association between the upper respiratory tract Microbiota and the severity of COVID-19 remains to be elucidated. Therefore, further studies in this field are warranted to provide a comprehensive understanding of these relationships (58–60, 62, 405).

## 5 Conclusions

Humanity has undergone a protracted journey of nearly four years since the start of the COVID-19 pandemic, and fortunately, the landscape has undergone significant positive changes. Despite the WHO declaring the “end of COVID-19 as a public health emergency,” the virus persists, circulating and causing loss of lives while undergoing mutations, albeit to a lesser extent due to widespread vaccination and prophylactic measures. This underscores that SARS-CoV-2 remains a threat, albeit reduced from its earlier impact. Despite the wealth of current evidence and information, it remains uncertain whether the virus could stage a resurgence and evolve into an endemic or seasonal presence. Clinical and laboratory biomarkers have played a crucial role in assessing infection progression, enabling improved clinical management, and facilitating tailored therapies based on individual patient conditions (63–65). Although the fatality and infection rate of SARS-CoV-2 has decreased compared to the beginning of the pandemic and previous years thanks to the efficiency of vaccines, strains, and variants that are capable of avoiding this therapeutic measure still continue to emerge, which raises doubts about whether future booster doses are necessary despite the appearance of large epidemiological outbreaks of cases, and highlights the need to establish continuous surveillance systems against this pathogen and others with pandemic potential (66, 67). Currently, the most pressing concern lies in the aftermath of COVID-19, with common sequelae including pulmonary fibrosis, respiratory distress, pulmonary thromboembolism, damage to the epithelium, and embolisms. This underscores the need for a comprehensive understanding of the consequences of SARS-CoV-2 infection and the management of post-COVID complications (**Table 3**) (10).

In conclusion, this review presents a comprehensive overview of the factors leading to severe or chronic COVID-19. We have explored the disease from its pathophysiology, considering both viral and host factors, to the complications caused by SARS-CoV-2 infection, particularly in the context of the post-pandemic period of COVID-19. Additionally, this work emphasizes the importance of remaining vigilant to the circulation of variants and the overall impact of SARS-CoV-2 infection on the global population, especially in people with comorbidities or at high risk.

## Author contributions

JC: Writing – original draft, Writing – review & editing. VG-C: Writing – original draft, Writing – review & editing. SC: Writing – original draft. CC-V: Writing – original draft. CS-A: Writing – review & editing. AA: Writing – original draft. CM: Writing – review & editing. AR-D: Writing – review & editing. SB: Writing – review & editing. PG: Writing – review & editing. AK: Writing – review & editing. ML: Writing – original draft, Writing – review & editing.
